# Comment on: “Technical considerations in interpreting rapid transfusion through a 4.2-Fr Broviac catheter”—a reply

**DOI:** 10.1186/s40981-026-00881-5

**Published:** 2026-10-07

**Authors:** Shoko Fujioka, Yusuke Miyazaki, Hinako Furuya, Chika Miyazaki, Nobuyuki Katori, Yoshie Taniguchi

**Affiliations:** Department of Anesthesiology, The Jikei University School of Medicine, 3-25-8 Nishi-shimbashi, Minato-ku, Tokyo, 105-8461 Japan

**To the Editor,**

We thank Dr. Mizuno for his thoughtful letter [1]. concerning our case report [2]. We greatly appreciate the opportunity to provide the technical details requested.

Because the current case was an elective rather than an emergency procedure, a multidisciplinary meeting was held in advance, as described in our case report. In addition to the various healthcare professionals from the departments listed in the case report, representatives from our hospital’s Patient Safety Committee participated in the preoperative conference. The conference addressed not only strategies for rapid transfusion but also the overall safety management of the anticipated acute hemorrhage, including alternative surgical approaches. When the participants discussed the use of the Broviac™ catheter for rapid transfusion, they confirmed that its intraluminal pressure should not exceed 25 psi (approximately 1,293 mmHg) according to the manufacturer’s instructions. Given this limit and the high pressures that manual syringe injection can generate [3], a consensus was reached that syringe-driven rapid transfusion through the Broviac™ catheter would be inappropriate. In contrast, the SL One^®^ continuously monitors in-line pressure and is designed to adjust the rotation speed of its roller pump automatically so that the in-line pressure does not exceed 300 mmHg. The conference participants therefore concluded that rapid transfusion through the Broviac™ catheter using the SL One^®^ was a reasonable approach, and the device was selected for this case.

The flow rate of 150 mL/min described in our report was the programmed rate. Because the pump was automatically slowed to limit the in-line pressure, the maximum delivered flow rate was 126.8 mL/min, at which the in-line pressure was 291 mmHg (approximately 5.6 psi). The highest in-line pressure was 331 mmHg (approximately 6.4 psi), which occurred transiently at a delivered flow rate of 93.8 mL/min. The device immediately reduced the flow rate, and the in-line pressure then decreased to 50 mmHg. Thus, although the in-line pressure transiently exceeded 300 mmHg, the automatic pressure-regulation mechanism of the SL One^®^ kept the in-line pressure far below the catheter manufacturer’s limit throughout the transfusion.

As Dr. Mizuno pointed out, catheter length is one of the determinants of in-line pressure. An accurate record of the total catheter length was not available for the present case. However, the in-line pressure reflects the resistance of the entire circuit, of which the catheter is only one component. Because the SL One^®^ continuously monitors the in-line pressure during use, it allows transfusion to be performed at pressures far below the catheter manufacturer’s limit, regardless of catheter length. After the rapid transfusion, the Broviac™ catheter showed no evidence of leakage or structural damage and continued to function normally throughout the postoperative period.

We emphasize that this approach was adopted in an elective setting after multidisciplinary preoperative planning. Our experience suggests that the SL One^®^ may enable rapid transfusion through a Broviac™ catheter while allowing continuous monitoring and automatic adjustment of in-line pressure. Further clinical experience is needed to establish the safety and generalizability of this approach.

## Data Availability

The data used in this report are available from the corresponding author upon reasonable request.
